# Migraine Information on the Web for Patients: A YouTube Content Analysis Based on a Scoring System

**DOI:** 10.7759/cureus.51054

**Published:** 2023-12-24

**Authors:** Rajat Gupta, Rajeswar Kumar, Dharma Teja, Geetanjali Kadiyala, Pallak Gautam, Manav Khalatkar

**Affiliations:** 1 Internal Medicine, Amar Hospital, Patiala, IND; 2 Medicine, Rajah Muthiah Medical College, Chidambaram, IND; 3 Internal Medicine, Mamata Medical College, Khammam, IND; 4 Internal Medicine, Government Medical College, Ongole, Ongole, IND; 5 Internal Medicine, Emilio Aguinaldo College, Manila, PHL; 6 Internal Medicine, Government Medical College and Hospital, Nagpur, Nagpur, IND

**Keywords:** discern, gqs, content analysis, youtube, headache, migraine

## Abstract

Introduction

Migraine, as a major cause of headaches, showcases the need for the public to be well aware of it. The legitimacy and quality of YouTube as a platform to find information regarding migraine have not been validated. The aim of this study was to assess the content, quality, and reliability of information about migraine on YouTube.

Methodology

Videos were reviewed on YouTube after searching for relevant keywords. They were screened for a predetermined inclusion criterion and they entered into a performed questionnaire by authors. Using the Global Quality Scale and DISCERN scale, the effectiveness of the videos was evaluated. These videos were further analyzed for viewership and their relation to the effectiveness of the videos by the Video Power Index (VPI).

Results

The videos published by “others” had the highest VPI, at 517.13, followed by videos uploaded by doctors, at 117.91. The difference in the VPI was determined to be statistically significant across various groups (p=0.033). The doctors’ videos received the highest reliability ratings, but the difference between them and “others” was not statistically significant (p=0.317).

Conclusions

Videos regarding migraine on YouTube could be more effective. The latest preventive strategies must be provided, together with supporting evidence from the authors.

## Introduction

Migraine is one of the leading causes of headaches among individuals aged 15-49 years worldwide, causing considerable distress [1,2]. Although the high prevalence of migraine might suggest widespread awareness, knowledge about migraine in the general population appears to be limited [3,4].

In the last decade, the most common online sites for gathering information have been YouTube and Facebook, with a major portion of the adult population having access to the internet [5]. There has been significant growth in interest in using technological advancements in the diagnosis and treatment of migraine headaches [6]. Recent years have seen an increase in interest in studying the non-pharmacological modes of treatment of migraine headaches, such as exercise and meditation, which have been shown to have fewer side effects compared to pharmacological interventions [7].

Only a minor portion of the videos regarding migraine treatment are authorized by healthcare professionals, whereas the majority of the videos are from non-healthcare advisors, which might affect the quality of the treatment received by patients following online platforms [8]. This might discourage patients from making a hospital visit upon an attack or seeking treatment afterward [9]. Many users access this information on YouTube and various other search engines to search for information about the cause and remedy for their condition or simply out of curiosity [10].

This study aimed to assess the perception and prevalence of the use of YouTube as a source of information for medical knowledge, helping both migraine patients and learners.

Aims and objectives

The aim of this study was to assess the quality and reliability of the information available on YouTube about migraine. The assessment was made using GQS (Global Quality Scale) and DISCERN (Quality Criteria for Consumer Health Information).

## Materials and methods

Study design and data collection

This research adopts a cross-sectional observational study design, conducted in June 2023, to explore the landscape of migraine-related information available on YouTube. To ensure a comprehensive analysis, a specific questionnaire was meticulously crafted by the authors and made accessible online via Google Forms. This questionnaire incorporated predetermined criteria, thoughtfully selected by the authors to guide the study's direction.

Data retrieval and search strategy

The initial step involved the retrieval of YouTube content for analysis. A systematic search strategy was employed, leveraging a set of keywords relevant to migraine, including "Migraine," "migraine symptoms," "migraine treatment," "migraine headache," "migraine exercise," "stop migraine," and "migraine attack." These keywords served as the compass to navigate the vast sea of available videos, ensuring that the search was comprehensive and informative.

Video assessment and selection

To maintain rigor and objectivity in the study, a team of six authors actively participated in the video assessment process. Each author reviewed and assessed 10-15 YouTube videos, culminating in a total dataset comprising 83 videos. This comprehensive set of videos was strategically chosen to encompass a wide spectrum of information, including aspects of migraine symptomatology, treatment modalities, etiology, diagnostic investigations, preventive strategies, mortality statistics, and rehabilitation methods.

The videos that made it into the final analysis underwent a meticulous selection process based on predefined inclusion criteria. The inclusion criteria included the following factors: relevance to the topic of migraines, ensuring that videos provided substantial and accurate information regarding migraines; language, selecting videos presented in either English or Hindi to accommodate a diverse audience; duration, considering videos with a duration ranging from 1 to 20 minutes to ensure the inclusion of both concise overviews and more detailed presentations.

Assessment of video quality and reliability

The quality and reliability of the selected videos were subjected to a rigorous evaluation process using two established assessment tools: the GQS and the DISCERN (Quality Criteria for Consumer Health Information) [11,12]. These tools provided a structured framework for evaluating various aspects of the videos, such as the accuracy of information, clarity of presentation, and overall quality.

Statistical analysis

Data analysis was conducted using the IBM SPSS Statistics for Windows, Version 21 (Released 2012; IBM Corp., Armonk, New York), a robust software widely utilized for statistical analysis in research. This enabled the generation of meaningful insights and statistical findings from the collected data.

In summary, the methodology encompasses a comprehensive and systematic approach to analyzing the wealth of information available to migraine patients on YouTube. By employing a carefully designed questionnaire, conducting thorough video searches, and applying diligent evaluation criteria, the study aims to provide a comprehensive assessment of the quality and reliability of online migraine-related content.

## Results

A total of 83 videos on migraine were initially selected, but after applying the inclusion/exclusion criteria, about 70 videos were taken into consideration.

Of the 70 videos, 51 (73.9%) were uploaded more than a year ago. Hospital YouTube channels led with 17 (24.6%) videos, followed by doctors with 16 (23.2%). The videos had 304,718,805 views and 51,831 comments in total (Table 1).

**Table 1:** Characteristics of YouTube videos analyzed

Time since uploaded	
More than a week to six months (7-180 days old)	6 (8.7%)
More than six months to last one year (180-365 days)	12 (17.4%)
More than one year (>365 days)	51 (73.9%)
Popularity	
Total no. of views	30,471,805
Total no. of likes	641,182
Total no. of dislikes	15,848
Total no. of comments	51,831
Type of uploader	
Doctor	16 (23.2%)
Hospital	17 (24.6%)
Healthcare organization	7 (10.1%)
News channel	14 (20.3%)
Other	15 (21.7%)

About 47 (68.12%) of the videos discussed etiology, whereas 57 (82.61%) discussed migraine symptoms. The topics of prevention and treatment were covered in 39 (56.52%) and 38 (55.07%) videos, respectively. Only 13 (18.84%) of the videos mentioned the latest developments in migraine treatment. Only 2 (2.9%) videos provided information regarding migraine support groups, and 8 (11.59%) featured patients or people talking about their own experiences with migraine (Table 2).

**Table 2:** Information on migraine in the YouTube videos

Information collected from the videos	No. of videos relaying the information
Description of symptoms of migraine	57 (82.61%)
Information about cause/etiology?	47 (68.12%)
Information about investigation and tests	8 (11.59%)
Information about prevention/vaccines	39 (56.52%)
Information about treatment	38 (55.07%)
Information about mortality	6 (8.7%)
Information about rehabilitation	14 (20.29%)
Information about support groups	2 (2.9%)
Information about patients sharing their own experience	8 (11.59%)
Information about people sharing experiences with their family members	2 (2.9%)
Information about recent advances in treatment	13 (18.84%)
Does the post have promotional content by pharmaceutical companies or by doctors?	10 (14.49%)

Based on the type of uploader, there was a significant difference in the Video Power Index (VPI) with a P-value of 0.033 (Table 3). However, there was no significant difference in the quality and reliability of videos uploaded by different users.

**Table 3:** Comparison of GQS, reliability score, and VPI based on the type of uploader GQS: Global Quality Scale, VPI: Video Power Index

	Doctors (n=16)	Hospital (n=17)	Healthcare organization (n=7)	News channel (n=14)	Others (n=15)	P-value and test used
	Median (IQ1, IQ3)	Median (IQ1, IQ3)	Median (IQ1, IQ3)	Median (IQ1, IQ3)	Median (IQ1, IQ3)	Test Used: Kruskal-Wallis test
VPI	117.91 (41.35, 441.18)	47.05 (6.75, 289.16)	30.01 (14.19, 94.13)	63.11 (18.88, 381.04)	517.13 (123.67, 1361.91)	P-value = 0.033
GQS	4 (3.25, 5)	5 (4, 5)	4 (2, 4)	4.5 (2.75, 5)	3 (3, 5)	P-value = 0.060
Reliability score	4 (3, 4)	3 (3, 4)	3 (3, 4)	3 (3, 4)	3 (3, 4)	P-value = 0.317

## Discussion

Our study aimed to investigate the characteristics of YouTube videos on migraine and their potential impact on people with migraine. After a thorough analysis of the data collected, we gained insight into several factors associated with the characteristics of the videos, including viewership, reliability and quality, the nature of the uploader, and the motivation behind the videos. The study demonstrates that YouTube is a major source of information for people seeking information regarding migraine and associated symptoms.

The majority of the videos (73.9%) in our study were posted more than a year ago, with the total number of views for all videos combined being more than 30 million. This indicates that a substantial pool of information regarding migraine is available, and the high number of likes (641,182) and comments (51,831) demonstrates the wide reach and impact of YouTube as a source of information on migraines. Therefore, it becomes very important that the information is reliable and accurate. Most of the videos were posted by either a doctor (23.2%) or a hospital (24.6%), with significant contributions from news channels and other sources, which shows the engagement of medical professionals in reaching out to the public with reliable information. In a similar study by Saffi et al., most of the videos (59%) were uploaded by the “other” group with a total combined view count of more than 163 million for 607 videos [13]. The reason for this might be because their study was conducted in the United States, where many people consider looking for "complementary and alternative medicine" for migraine (which were grouped under the “other” category) due to a lack of insurance, high cost of healthcare, and other reasons.

The main content for about 82.61% of the videos in this study is about the description of migraine symptoms and their various presentations, with 68.12% and 56.52% of the videos providing information regarding the etiology and prevention measures respectively. The major focus of these videos on describing the symptoms and preventive measures shows that the main goal was to provide information and aid the patients to be better prepared to identify and tackle migraine attacks. The amount of promotional content was identified in only about 14.49% of the videos. This further confirms that the major purpose of most of the videos was for the benefit of the patients and the general population to provide evidence-based information to enable them to make informed decisions. In contrast, a study by Yuce et al., on YouTube information for patellofemoral instability (PFI), mentions that patients with pre-information from YouTube make the consultation with a healthcare professional less efficient and affects the decision-making process compared to an otherwise normal consultation [14]. However, PFI is a complex condition to which the explanations provided on YouTube might not suffice leading to such problems. Still, this should be one area of concern where pre-information from YouTube might affect the consultation and decisions with a healthcare professional. In addition, only a very small percentage of videos talk about the recent advances in treatment (18.84%), support groups (2.9%), and people sharing their own experiences (2.9%). Therefore, we urge future uploaders to consider focusing on these less discussed aspects of migraine management.

The VPI is a value that is derived as a product of the like ratio and the view ratio divided by 100 [14]. The statistical analysis of the results obtained from this study using the Kruskal-Wallis test, VPI was found to be the highest in the videos uploaded by ‘others’ with a VPI of 517.13 followed by the videos uploaded by doctors with a VPI of 117.91 and the difference in VPI was found to be statistically significant among the different groups (p = 0.033). This reflects that the videos uploaded by “others” have a higher view count compared to those by doctors or hospitals. It may be understood that the videos uploaded by “others” are better understood and relatable to the public and, hence are well received. To assess the quality and reliability of the videos, the Global Quality Scale (GQS) and the DISCERN tool were used [15]. The GQS was found to be the highest in the groups of hospitals and news channels with a score of 5 and 4.5, respectively. But the difference was not found to be statistically significant between the groups (p = 0.06). The reliability score was the highest in the videos by doctors and the difference was not statistically significant between the different groups (p = 0.317). This finding demonstrates that the quality and reliability of the information provided in the videos were comparable across various uploaders and it is reassuring that the uploaders did not compromise on the quality of their videos irrespective of their professional affiliation.

Limitations

Since this is a cross-sectional study, the data was collected at one point in time. While YouTube is a dynamic platform, its features and the data collected get updated every second. This study only included videos available in English and Hindi, which could cause potential bias since India is a country with several regional languages, and the videos uploaded in such regional languages were not considered in the study. Healthcare practitioners can contribute to improving the quality and reliability of YouTube videos and better serve people looking for information on migraines by addressing these limitations and building on the results of the study.

## Conclusions

YouTube is a vast source of information for migraine-related queries, which could educate patients on when and how to manage their condition independently when appropriate and seek professional help when needed. Overall, this study shows that there is a wide range of information available on YouTube about migraine. This presents both opportunities and challenges to ensure that the available information is accurate, reliable, and evidence-based. For this purpose, future collaborations between medical professionals, researchers, and content creators can play a vital role in promoting accurate, reliable, and up-to-date information, ultimately for the benefit of the people seeking information on migraine. 
